# Development and Evaluation of an HIV-Testing Intervention for Primary Care: Protocol for a Mixed Methods Study

**DOI:** 10.2196/16486

**Published:** 2020-08-17

**Authors:** Hanne Apers, Bea Vuylsteke, Jasna Loos, Tom Smekens, Jessika Deblonde, Dominique Van Beckhoven, Christiana Nöstlinger

**Affiliations:** 1 Department of Public Health Institute of Tropical Medicine Antwerp Belgium; 2 Centre for Migration and Intercultural Studies University of Antwerp Antwerp Belgium; 3 Domus Medica Antwerp Belgium; 4 Faculty of Medicine and Health Sciences University of Antwerp Antwerp Belgium; 5 Epidemiology of Infectious Diseases Unit Department of Public Health and Surveillance Belgian Scientific Institute of Public Health (Sciensano) Brussels Belgium

**Keywords:** primary care, general practitioners, public health, HIV testing, Belgium, intervention

## Abstract

**Background:**

Late diagnosis of HIV fosters HIV transmission and may lead to hidden HIV epidemics. In Belgium, mathematical modeling indicates a high prevalence of undiagnosed HIV infections among men who have sex with men of non-Belgian origin and among sub-Saharan African migrants. Promotion of HIV testing facilitates early diagnosis, but diagnostic opportunities are missed in primary care.

**Objective:**

The intervention study aims to enhance provider-initiated HIV testing by GPs. This protocol presents the conceptual development, implementation, and evaluation of an HIV-testing intervention for Flemish general practitioners (GPs).

**Methods:**

A mixed methods evaluation design is used. Guided by a simplified intervention mapping approach, an evidence-based intervention was developed in collaboration, guided by an interdisciplinary advisory board. The intervention consisted of an evidence-based tool (ie, “HIV-testing advice for primary care”) to support GPs in provider-initiated HIV testing. A modified stepped-wedge design compare two different intervention levels: (1) online dissemination of the HIV-testing advice and (2) dissemination with additional group-level training. Both conditions were compared against a control condition with no intervention. The effect of the intervention was measured using Poisson regression for national surveillance data. The primary outcome was the number of HIV diagnoses made by GPs. Secondary outcomes were HIV diagnoses among groups at risk for undiagnosed HIV, distribution of new diagnoses by CD4 cell count, number of HIV tests prescribed by GPs, and rate of new diagnoses by tests. To evaluate the intervention’s implementation, the GPs’ fidelity to the intervention and the intervention’s feasibility and acceptability by GPs were assessed through (web-based) surveys and in-depth telephone interviews.

**Results:**

The study was funded in 2016 and ethically approved in January 2017. The implementation of the intervention started in January 2017 and ended in December 2018. Data was completed in October 2019 and was the starting point for the ongoing data analysis. The results are expected to be published in the second half of 2020.

**Conclusions:**

Results of the intervention study will provide useful information on the intervention’s effectiveness among Flemish GPs and can inform further development of official testing guidelines. Limitations of this real-life intervention approach are potential spill-over effects, delay in access to surveillance data, and little detailed information on HIV-testing practices among GPs.

**Trial Registration:**

ClinicalTrials.gov NCT04056156; https://clinicaltrials.gov/ct2/show/NCT04056156

**International Registered Report Identifier (IRRID):**

DERR1-10.2196/16486

## Introduction

HIV-positive individuals who are unaware of their status have a higher risk of transmitting HIV [1], potentially fueling a hidden HIV epidemic. HIV testing is a crucial step in HIV prevention continuum facilitating timely HIV diagnosis [2]. However, in 2017, about half of the HIV patients newly diagnosed in countries that belong to the European Union/European Economic Area with a known CD4 cell count were diagnosed late (CD4 cell count<350 cells/mm^3^); 28% were diagnosed at an advanced stage (CD4 cell count<200 cells/mm^3^) [3]. In Belgium, two key populations are most affected: men who have sex with men (MSM) and sub-Saharan African Migrants (SAMs) [4]. More than a third of new Belgian HIV cases were diagnosed late in 2017, ranging from 27% among MSM to 50% among SAMs.

The *HIV European Research on Mathematical Modeling and Experimentation of HIV Testing in Hidden Communities* (HERMETIC) project applies a mathematical model for routine HIV-surveillance data to calculate the size and proportion of these undiagnosed HIV-infected populations. Modeling results obtained using a back-calculation method [5] for Belgium indicate that almost 2818 people living with HIV remained undiagnosed in 2015, corresponding to a prevalence rate of 4.1 undiagnosed persons per 10,000 [6]. The most affected populations were non-Belgian MSM (almost 264 undiagnosed persons per 10,000), SAMs with a higher proportion among women (about 173 per 10,000) than among men (about 93 per 10,000), and Belgian MSM (about 55 per 10,000) [6]. While Belgian HIV-testing rates are relatively high and cost-effective with 1.25 new HIV diagnoses per 1000 tests [4,7], these estimates of undiagnosed HIV infections indicate that current HIV-testing strategies fail to reach all people at risk of infection in a timely manner.

General practitioners (GPs) are well placed to facilitate early HIV diagnoses; their long-term and holistic relationships with patients provide opportunities for HIV testing, as they are the first entry point to health care [8-10]. However, many diagnostic opportunities are missed in primary care [11-13]. A recent systematic review showed the following GP-specific barriers and facilitators: lack of time, fear to disturb the consultation process, language barriers, lack of culture-sensitive sexual counseling skills, and concerns about pretest discussion or result management [11]. GP-specific training and practical tools to support HIV testing were found to facilitate HIV testing in primary care. Earlier research mentions similar barriers among Belgian GPs [14]. A recent analysis on Belgian surveillance data has shown that Belgian GPs predominantly diagnose MSM, whereas SAMs are more often diagnosed by specialists [15]. The fact that about half of all HIV tests conducted in Belgium in 2017 were prescribed by GPs [4] and that complementary community-based testing initiatives are in place [16,17] indicates the potential to increase the HIV-testing offer in primary care.

**Figure 1:**
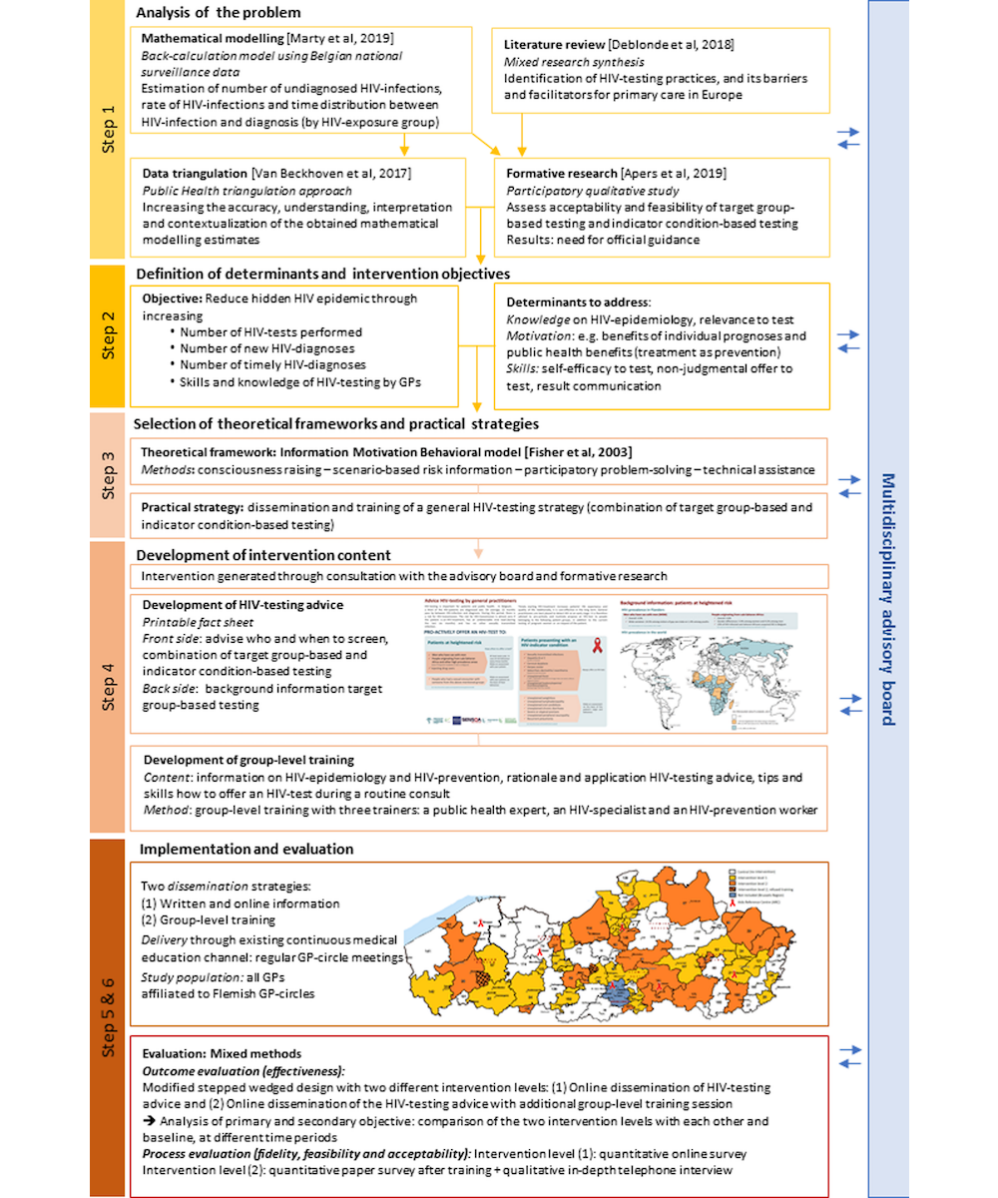
Developing an HIV-testing intervention for Flemish GPs. A stepwise approach to intervention development 19.

To identify solutions to these barriers and reduce the hidden HIV epidemic in Belgium, we developed an HIV-testing intervention for primary care by adopting a simplified intervention mapping approach [18,19] (Figure 1). This approach was chosen to ensure appropriateness and future sustainability, as it promotes the use of theory and evidence, as well as the inclusion of stakeholders [18]. Collaborative participation [20] was ensured through an advisory board consisting of 22 experts including GPs, representatives of GP umbrella organizations, policy makers, HIV care specialists, public health specialists, prevention specialists, and lab specialists. The advisory board has been consulted at all stages of the research, providing inputs to content and strategic decisions.

In the preparatory phase, a detailed contextual analysis of the problem within the local Flemish context was conducted (see step 1 in Figure 1). The aforementioned modeling results [6] were triangulated with multiple data sources [21], such as behavioral surveys and an expert meeting for validation and to gain further in-depth insight. The results of a mixed research synthesis on HIV testing in European general practices [11] provided additional guidance. The advisory board selected two evidence-based HIV-testing strategies to be further elaborated for this intervention: provider-initiated testing of key populations and indicator condition–based testing. A participatory, formative research with 122 GPs using group discussions was conducted to obtain their inputs on the acceptability and feasibility of these strategies [22]. While the two proposed testing strategies were generally accepted by the participants, deficits in knowledge about relevant HIV epidemiology and skills to propose testing became apparent. GPs emphasized on the lack of official policy guidelines to support them in provider-initiated HIV testing.

On the basis of the results of the preparatory phase, it was decided in collaboration with the advisory board that the intervention should integrate the two assessed HIV-testing strategies into one intervention consisting of a GP-specific HIV-testing tool for providing information and an accompanying group-level training that practically supports GPs in performing provider-initiated HIV testing. Here, we present the HERMETIC study protocol for the intervention’s content development, and the practical strategies for implementation and evaluation in Flanders, Belgium (steps 2, 3, 4, 5, and 6 in Figure 1).

## Methods

**Figure 2:**
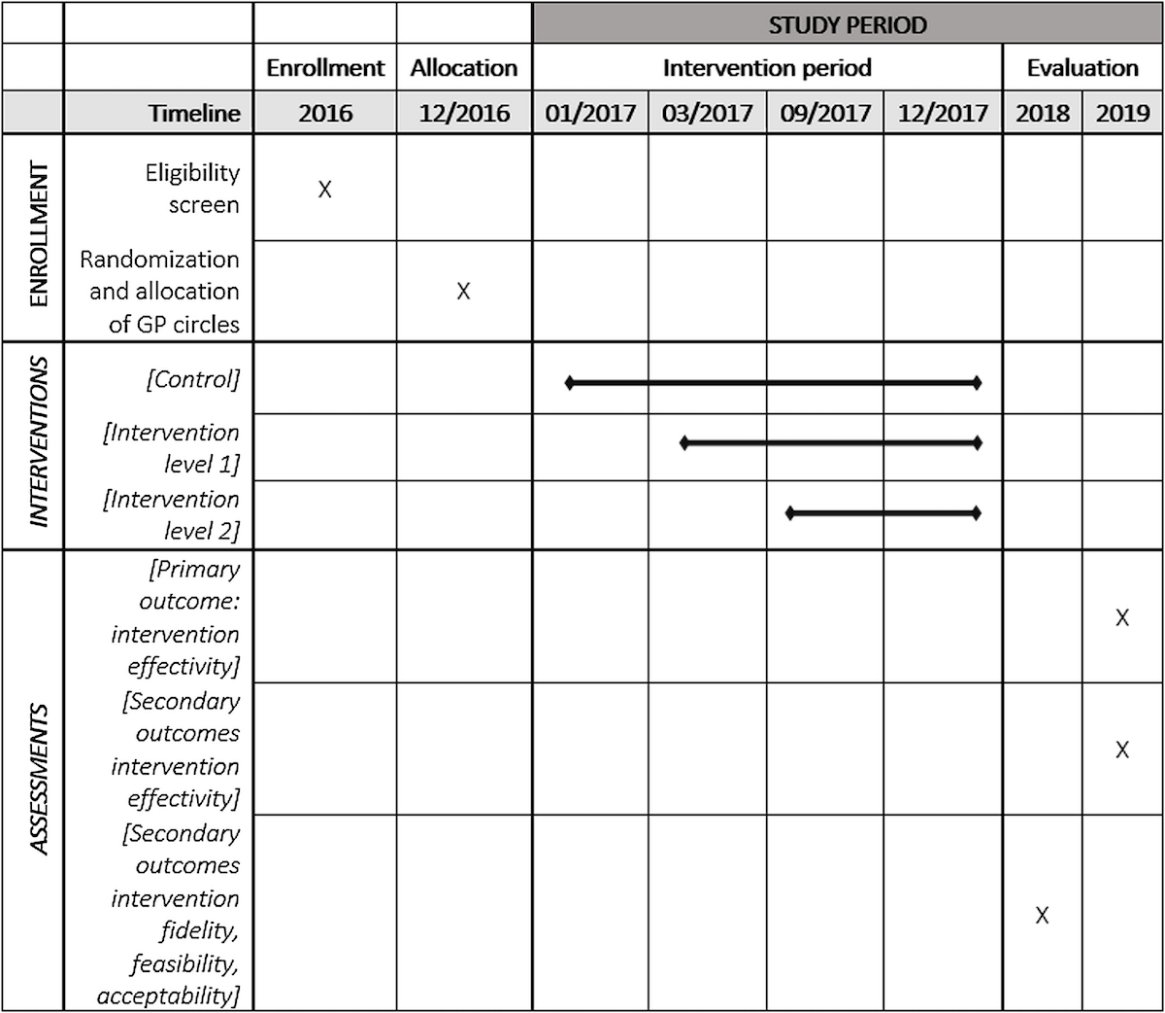
Schedule of enrolment, allocation, intervention, and evaluation of the HIV-testing intervention.

Figure 2 shows a schedule of the enrollment, intervention, and evaluation of the HIV-testing intervention.

### Objectives and Outcomes

The objective of the study is to evaluate the effectiveness of two different intervention levels (dissemination of the information tool and group-level training) against a control condition. This will be assessed by the following primary and secondary outcomes:

1. Primary outcome measure is the number of HIV diagnoses made by the GPs. We will evaluate the change in the number of new HIV diagnoses made by GPs by comparing the data from 2016, which serve as baseline data, to data from 2017 and 2018.
2. Secondary outcome measures are HIV diagnoses among groups identified to be more likely undiagnosed, distribution of new diagnoses by CD4 cell count (to assess changes in late HIV diagnoses), the overall number of HIV tests prescribed by GPs, and the rate of new diagnoses by the number of HIV tests performed.

To evaluate the intervention’s implementation, GPs’ fidelity to the intervention and its perceived feasibility and acceptability by GPs are assessed using mixed methods (see “Methods” section).

### Study Population

The study aims to enroll all Flemish GPs who are affiliated to a *“GP circle” (ie, huisartsenkring or HAK)*. The *HAK* is the local focal point for GPs and policy makers to implement health policy in primary care, and provides continuous medical education [23]. HAKs are subsidized by the Flemish government and are organized by geographical zones. In 2016, 8163 GPs were active in Flanders [24]. Among them, 6211 (76.1%) are affiliated to one of the 86 HAKs (Zorg en Gezondheid, personal communication via email, September 2016). The choice of enrolling only GPs affiliated to a HAK is a pragmatic one: GPs can be easily accessed through HAKs, and the geographical aspect provides an opportunity for nonindividual comparison. No data are available to describe GPs who are not affiliated to HAKs, but they are assumed to be mainly working in nonprimary care contexts such as specialist centers for educational health care (Zorg en Gezondheid, personal communication via email, September 2016).

### Intervention Content and Practical Strategies

The findings of HIV knowledge gaps and testing skills as determinants among GPs during the formative research [22] led to the selection of the empirically widely tested i*nformation-motivation-behavioral skills model* [25] as a theoretical base for the intervention’s content, practical strategies, and implementation (see steps 2 and 3 in Figure 1). To meet GPs’ demand for official guidelines, the intervention should include dissemination of an HIV-testing advice and a group-level training (step 4 in Figure 1).

#### HIV-Testing Advice

**Figure 3:**
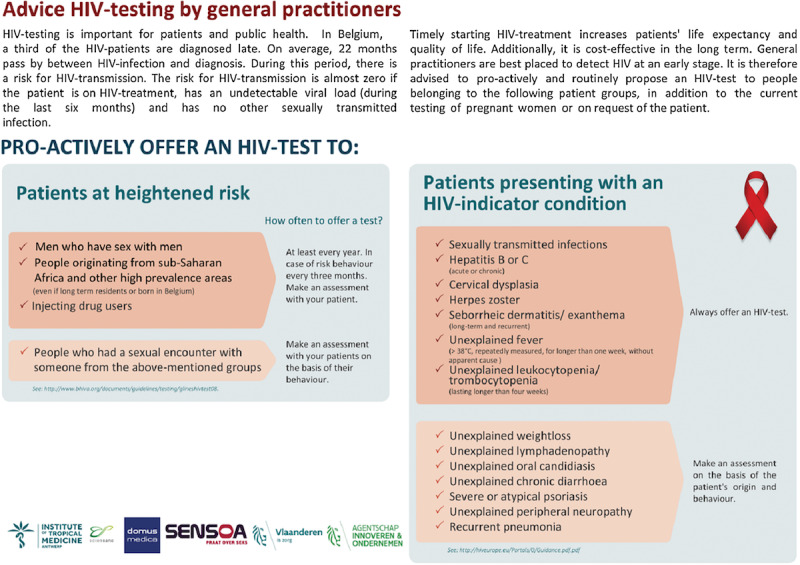
Advice HIV-testing by GPs (front side).

**Figure 4:**
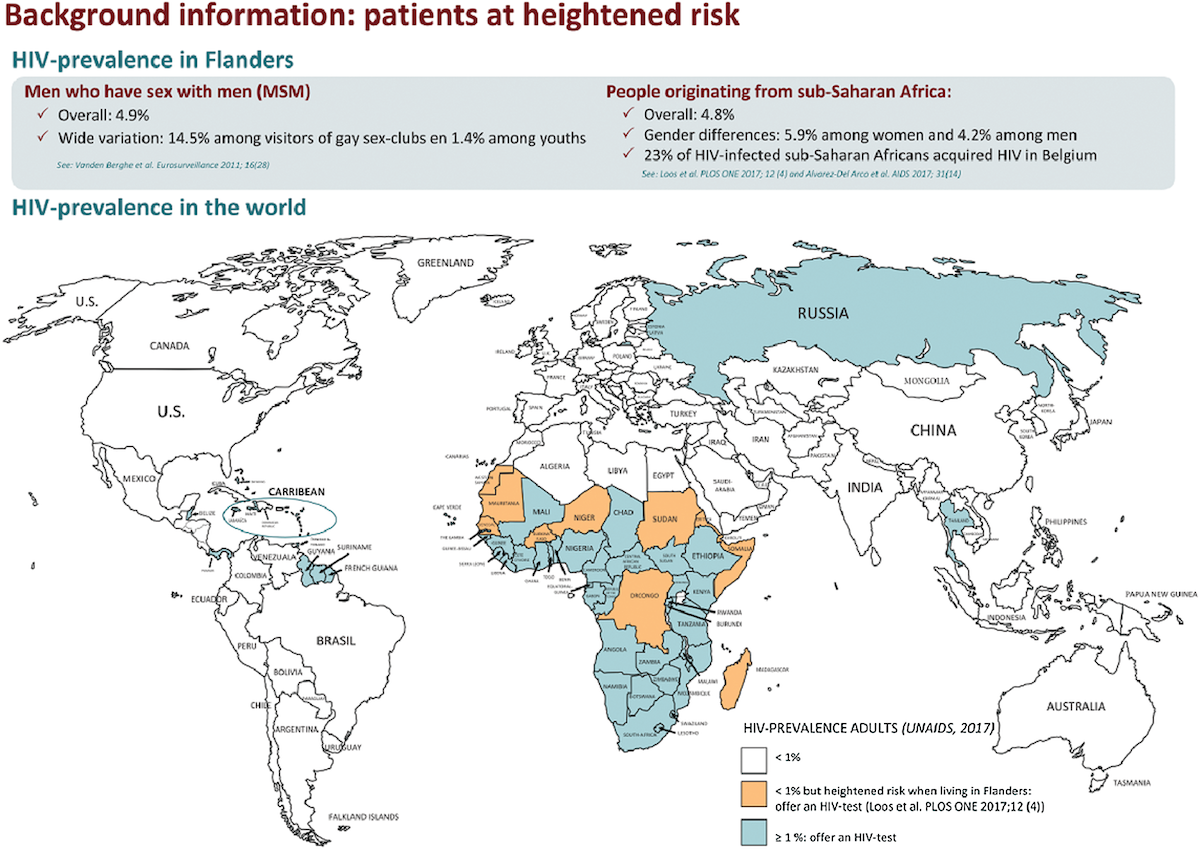
Advice HIV-testing by GPs (back side).

User-friendly advice combining the two selected HIV-testing strategies was created (see Figures 3 and 4). Informed by the expressed needs of GPs during the formative research, it guides them in proactively testing patients at heightened risk of HIV acquisition and patients presenting with an HIV-indicator condition and gives a time indication of how often an HIV test should be performed. National epidemiological information and the HERMETIC modeling results have defined the key target groups that an HIV test should be offered to. The extensive 64 HIV-indicator conditions formulated in the HIV Indicator Diseases Across Europe Study [7] were reduced to 14 conditions often diagnosed in primary care, as indicated by the formative research results and in collaboration with the AB [22]. The advice also provides country-specific information on HIV prevalence as an additional rationale as to why targeting patients at heightened risk due to migration from high endemic regions is recommended.

#### Group-Level Training

GPs expressed their preference for a face-to-face training as an intervention delivery channel [22]. To increase GPs’ motivation and skills on HIV testing, we designed a group-level training for 1.5-2 hours, consisting of three main parts, each led by trainers with different types of expertise. The introduction given by a public health expert discusses current HIV epidemiology, advantages of early diagnosis, missed opportunities in primary care, current HIV-prevention approaches and GPs’ role in these, and common misconceptions about HIV risk. The second part presents the HIV-testing advice in detail. An HIV specialist provides specific medical information (ie, on the indicator conditions). The third part given by an HIV-prevention worker focuses on the use of the advice including aspects of patient-provider interaction and communication skills. In the latter part, specific tips and reference to hands-on tools were given to overcome the barriers related to the specific target groups (such as using the visual online support tool Zanzu [26] to reduce language- or culture-related barriers).

### Intervention Implementation

Two intervention levels were established, differing in terms of degree of information delivered and delivery channels. For the latter, the existing GP channels were selected: the website of the GP umbrella organization [27] and HAK networks. At the third level, the control condition, GPs receive no intervention, that is, they are not actively targeted according to the current standard practice.

#### Intervention Level 1: Online Dissemination of the HIV-Testing Advice

GPs included at the first level receive the HIV-testing advice through an email by their HAK coordinator, which contains an information message with the printer-friendly version of the HIV-testing advice attached (see Figures 3 and 4). The message also provides a link to the website of the Flemish umbrella organization for GPs, where the tool is available for download for all Flemish GPs. A reminder is sent out to all participants after 13 months.

#### Intervention Level 2: Online Dissemination of the HIV-Testing Advice with Additional Group-Level Training Session

At the second intervention level, GPs first receive intervention condition 1 and then the face-to-face group-level training session. These sessions are organized as part of regular continuous medical education provided by HAKs at their usual venues and are organized a few months after receiving intervention level 1. A reminder of the advice is sent out 13 months after the initiation of intervention level 1.

### Study Duration

Study duration including intervention development is from January 2017 to December 2019. Intervention implementation and evaluation take place in the framework of the HERMETIC study; implementation started in December 2017 and evaluation results will be available in December 2019 (see steps 5 and 6 in Figure 1).

### Study Design

This mixed methods study adopts a modified stepped-wedge design. The classical stepped-wedge cluster design involves a random and sequential crossover of clusters from control to intervention until all clusters are exposed. It includes an initial period in which no clusters are exposed to the intervention [28]. We adopted a modified version: the two different intervention levels are added stepwise, while a control condition is retained until the end of the study period. Participants of both intervention levels serve as their own (historical) controls receiving no intervention during approximately 2 months, then all the participants receive the HIV-testing advice online. Those assigned to the first intervention level remain at this level until the end of the project. Participants assigned to the second intervention level are offered the additional group-level training after 7 months. The rationale for this adaptation is twofold: (1) to compare the effects at all intervention levels among each other over time and (2) to assess feasibility (ie, resource and time restrictions).

The process of information distribution is monitored: confirmation is obtained through short phone interviews with GP-circle coordinators to verify if the emails were sent to individual GPs and to allow for timely assessment of problems and reasons for noncompliance with the procedure.

#### Randomization Strategy

Intervention groups are identifiable in HIV-surveillance data using the administrative code referring to geographical zones defined by the National Institute of Statistics (ie, NIS code). HAKs that correspond to the same NIS code have been merged, regrouping 86 HAKs into 76 clusters. Each cluster is randomly allocated by the study team to one of the three intervention conditions using block sampling. Block sampling took the variability of the different target populations at risk for HIV in the intervention groups into consideration: each block consists of three clusters with approximately the same number of SAMs in the respective NIS-code areas. The main reason for this approach is that the intervention is assumed to generate the largest effect among SAMs, given their low testing rates in primary care. Since SAMs are unevenly distributed over Flanders with the largest communities living in central cities [29], block randomization assures that the expected effect is equally distributed across the three intervention levels. This approach results in 25 clusters being assigned to intervention level 1 or 2, and 26 clusters assigned to the control condition.

### Intervention Evaluation

A mixed methods evaluation approach is applied throughout the intervention implementation (see “Objectives and Outcomes” section and steps 5 and 6 in Figure 1). An outcome evaluation assesses the intervention’s effectiveness and changes in GPs’ targeting behavior in HIV testing using routine surveillance data on new HIV diagnoses and HIV tests performed in Flanders. Changes in the number of new HIV diagnoses made by GPs, changes in the number of diagnoses among groups at high risk for undiagnosed HIV and at different stages of HIV infection, and changes in the number of HIV tests performed by GPs and in the rate of diagnoses by tests will be assessed.

Process evaluation assesses several implementation aspects: GP’s fidelity to the intervention in addition to the intervention’s feasibility and acceptability. In intervention level 1, this will be evaluated through an online survey sent out together with an email reminder. The survey is accessible for 2 months. In intervention level 2, GPs attending the group-level trainings are registered to calculate the actual attendance rate. Sociodemographic characteristics of the trained GPs and their evaluation of the training, perceived feasibility, and acceptability of the advise are assessed by means of a structured questionnaire.

A qualitative component collects in-depth and contextual insights on perceived fidelity to the intervention, feasibility, and acceptability of the group-level intervention. It serves to understand first-hand experiences with real-life implementation, for instance, if the intervention can be implemented as designed, and aims to better understand the intervention mechanisms. Data are collected among purposively selected, trained GPs for individual telephone interviews after 5-6 months. To guide this selection, potential “rich cases” among HAKs are identified considering specific indicators assessed through the questionnaire: appreciation of group-level training, anticipated feasibility and acceptability of the advice, and impact on knowledge and skills. HAKs with the lowest, middle, and highest median scores for each of the indicators are identified, and 10% of the trained GPs per HAK are randomly selected and invited to an interview. If GPs refuse, the reason for refusal is noted and another GP from the same HAK is invited until data saturation is obtained. No postprocess evaluation from patients is planned, as previous research has shown that proactively offering an HIV test is well accepted by patients [14].

#### Data Collection

For outcome evaluation, the following routine surveillance data are used:

- *National Health Insurance (Rijksinstituut voor Ziekte en Invaliditeitsverzekering [RIZIV]) data on the number of HIV tests conducted:* HIV tests are reimbursed by the Belgian National Institute for Sickness and Invalidity Insurance. Data on the number of HIV tests performed, the type of the physician requesting the test, and his/her geographical locations are available in the RIZIV database. The organization in charge of the HIV surveillance in Belgium (Sciensano, ie, the National Public Health Institute) receives extracted data on HIV tests prescribed by GPs, which will be aggregated according to the different clusters and intervention levels as identified by the NIS codes.
- *Laboratory data on new HIV diagnoses:* Seven national AIDS Reference Laboratories (ARL) perform HIV-confirmation tests on samples found to be reactive in screening tests in clinical laboratories. Yearly, each ARL reports data on all newly diagnosed HIV cases including sociodemographic, clinical, and limited behavioral characteristics (eg, transmission group). Sciensano links this information to the type of physician who initially prescribed the HIV test (collected through the physician’s registration number at the national social assurance system, ie, “RIZIV number”), the cluster number, and the intervention level (using the NIS codes of HAKs).

For process evaluation, data on perceived fidelity to the intervention, feasibility, and acceptability for intervention level 1 (information only) are collected through an online survey, located at the server of SoSciSurvey [30]. For intervention level 2, data are collected through a quantitative evaluation questionnaire immediately filled in on paper after the group-level training session. Data on experienced feasibility and acceptability are collected through recorded telephone interviews. In each of these data collected, limited sociodemographic characteristics of the participants are gathered, for example, age, gender, and years of experience.

#### Data Extraction and Management

For outcome evaluation, HIV-surveillance data will be transferred through technical interfaces with encrypted data to Sciensano by Healthdata, the agency responsible for data captation and secure data transfer. Data will be checked and duplicated records will be removed. Sciensano will provide the aggregated data of both HIV tests and HIV diagnoses to the researchers for further analysis.

For process evaluation, data from the online survey are collected anonymously and extracted through a downloadable Excel Database. Interview recordings will be transcribed verbatim. Pseudonymized data are managed and archived by the Institute of Tropical Medicine in locked and secured (digital) locations that are only accessible by the researchers.

#### Data Analysis and Statistical Methods

Using the stepped-wedge design, the distribution of results across unexposed observation periods is compared with that across the exposed observation periods. To determine the intervention’s effectiveness, primary and secondary outcomes at the three levels are compared against each other at three different time periods, that is, baseline and before March 2017, from March to August 2017, and from August 2017 to December 2018 (data available in late 2019).

Poisson regression is used to estimate the relative change in outcome variables. This change is explored through several alternative models, comparing the intervention group to the control group or to each other over time. In addition, we are investigating the differences in the ability to diagnose groups that are at high risk for acquiring HIV (MSM, SAMs, people who inject drugs, and others) and timely diagnosis (represented by CD4-cell count in three categories: less than 200 cells/mm^3^, 200-350 cells/mm^3^, and above 350 cells/mm^3^) across time periods and interventions.

To quantify the level of uncertainty associated with the estimates, Poisson regression models will be fitted to the data using Bayesian modeling under weakly informative priors. For each parameter, we will report the 90% central credible interval from the marginal posterior distribution.

Within the process evaluation, survey data are analyzed descriptively to compare groups with different intervention exposures in terms of self-perceived effects of the intervention levels such as gains in knowledge, self-efficacy to test, and future intention to use the intervention tools.

Qualitative data collected through telephone interviews are analyzed inductively. Using NVivo 8 (QSR International), a data-driven codebook will be established according to the principles of thematic analysis [31].

### Availability of Data and Materials

Datasets used and/or analyzed during the study are available from the corresponding author on request.

### Ethics and Dissemination

#### Informed Consent

Strict attention to maintain confidentiality is paid at every stage of data collection, analysis, and storage. Data extraction measures will be put in place to ensure that individual HIV patients cannot be identified (grouping of diagnosis as per HAK, analysis every 2 months). HIV-testing and diagnosis data will be managed through the legal entities in charge of the HIV/AIDS surveillance system (Royal Decree of October 8, 1996) authorized by the Belgian Privacy Commission.

To ensure transparency, GPs receive the HIV-testing advice, with an accompanying email that explains that the dissemination of the advice is part of the study monitoring the intervention’s effects through routine data collection. During the training sessions, GPs are informed of the same. The structured questionnaire for GPs contains an introductory statement with information stating that filling in the questionnaire is considered as providing informed consent.

Oral informed consent is obtained before the telephone interview from the participants, who agree to interviewed, in addition to their permission to audio-record the interview.

#### Dissemination

Results of the study will be disseminated at scientific conferences targeting GPs and HIV experts and through articles in GP-specific media and international peer-reviewed journals.

#### Ethical Approval

This study has been approved by the Institutional Review Board of the Institute of Tropical Medicine, Antwerp (approval number: 1228/18; dated January 6, 2017). The study is carried out according to the principles stated in the Declaration of Helsinki and according to established international scientific standards.

#### Consent to Participate

Not applicable for study effectiveness: analysis of HIV-testing and HIV-diagnosis data is managed through the legal entities in charge of the HIV/AIDS surveillance system (Royal Decree of October 8, 1996), authorized by the Belgian Privacy Commission. For transparency, GPs receive an accompanying email together with the HIV-testing advice, which explains that the dissemination of the advice is part of the study monitoring the intervention’s effects through routine data collection. During the training sessions, GPs are informed of the same.

With regard to the evaluation of the study’s feasibility, acceptability, and GPs’ fidelity to the advice, the structured questionnaire for GPs participating in the evaluation contains an introductory statement with information stating that filling in the questionnaire is considered as providing informed consent. Similarly, oral informed consent is obtained before the telephone interview from the participants, who agree to be interviewed, as well as their permission to audio-record the interview.

## Results

The study was funded in 2016 and was ethically approved in January 2017 (see Multimedia Appendix 1). The intervention implementation started in January 2017 and ended in December 2018. Data was completed in October 2019 and was the starting point for the ongoing data analysis. The results are expected to be published in the second half of 2020. The intervention study described in this protocol has been funded through the framework of the European HIV-ERA JTC 2014 (see Multimedia Appendix 2) and the Belgian Funding Agency IWT 140922. The Institute of Tropical Medicine is the study sponsor.

## Discussion

This study will deliver useful information on the implementation and effectiveness of an intervention to promote HIV testing among GPs in Flanders. The participatory intervention development strategy allows for a high degree of tailoring of the intervention to the needs of primary care providers. This should lead to a sustainable intervention [32] with high intervention effectiveness [33]. Systematically collected experiences on disseminating and adopting the HIV-testing advice can inform further development of national HIV-testing policies, sexual health strategies, and GPs’ respective roles in these. Yet, this approach of a real-life intervention trial has its limitations: spill-over effects among intervention levels may occur, as GPs not included in the respective intervention condition may download the advice from the GP umbrella organization’s website or may attend training events of a neighboring HAK. The influence of other initiatives to promote HIV testing cannot be excluded, for example, media messages on HIV testing or HIV prevention. Information on the background of GPs not affiliated to a GP circle is lacking and may cause a selection bias.

The use of surveillance data for analysis, however, provides an opportunity to evaluate a real-life intervention including all Flemish GPs. The formative research indicated this as the most feasible data collection method, enabling long-term follow-up while not overburdening busy GPs. However, such routine data are only collected once a year, therefore causing a delay in data access. Detailed information on specific HIV-testing conditions in primary care practices and profiles of testers cannot be collected. Privacy issues impede further subanalyses (eg, sociodemographic characteristics of GPs associated with diagnosis of HIV). In addition, other interventions, such as the introduction of preexposure prophylaxis (PrEP) on a national scale in June 2017 might cause changes in the demand for HIV testing and subsequently in the number of HIV diagnoses, making comparisons over time difficult.

This study provides a valuable contribution to evidence-based HIV testing in primary care, since only few interventions for the promotion of HIV testing in primary care have been described in the literature [11,34,35]. These settings may play a greater role in HIV prevention and care continuum [10] in the future with outlined directions in provider-level education interventions [35]. The study’s strong qualitative component will provide practice-based lessons on real-life experiences with HIV testing in primary care and insights into the mechanisms by which the intervention works. This will contribute to a nuanced interpretation of the results of outcome evaluation.

## Appendix

Multimedia Appendix 1 Approval letter Institutional Review Board of the Institute of Tropical Medicine.

Multimedia Appendix 2 Approval European framework HIV-ERA JTC 2014.

Multimedia Appendix 3 Previous peer-review report from the Institute of Tropical Medicine.

## Abbreviations

AB: advisory board
GP: general practitioner
HAK: HuisArtsenKring (GP-circle, local focal point for GPs)
HERMETIC: HIV European Research on Mathematical Modeling and Experimentation of HIV Testing in Hidden Communities
MSM: men who have sex with men
NIS: National Institute of Statistics
RIZIV: Rijksinstituut voor Ziekte en Invaliditeitsverzekering (Belgian National Institute for Sickness and Invalidity Insurance)
SAMs: sub-Saharan African migrants
