# Supplementary material for: Development and Evaluation of an HIV-Testing Intervention for Primary Care: Protocol for a Mixed Methods Study
Source: JMIR Res Protoc. 2020 Aug 17;9(8):e16486. doi: 10.2196/16486 (PMC7459432; doi:10.2196/16486)
Supplement: Multimedia Appendix 1 [file resprot_v9i8e16486_app1.pdf]

Institutional Review Board

Dr. Jasna Loos
Department of Public Health

IRB/AB/AC/057	OUR REF.	ATTACHMENT(S)	ANTWERP
	1228/18		24/09/2019

Concerns: HERMETIC Project: HIV-testing interventions for general practitioners in Flanders, Belgium; version 1.4, dated February 22, 2018

Dear Colleague,

After review at the IRB meeting of March 6, 2018, the above mentioned protocol has been approved.

Please kindly be reminded to send a yearly update report to the IRB (+ EC UZA) at the latest one year after the approval date.

The protocol does not need to be submitted to the EC of UZA for further review and approval.

Kind regards,

Dr. Raffaella Ravinetto

Chairperson Institutional Review Board
