# Supplementary material for: Development and Evaluation of an HIV-Testing Intervention for Primary Care: Protocol for a Mixed Methods Study
Source: JMIR Res Protoc. 2020 Aug 17;9(8):e16486. doi: 10.2196/16486 (PMC7459432; doi:10.2196/16486)
Supplement: Multimedia Appendix 3 [file resprot_v9i8e16486_app3.pdf]

| | |
|---|---|
| **IRB number** : | **1153/17** |
| **Title project/study:** | **European HIV-ERA research project 'HERMETIC': An HIV screening intervention for general practitioners in Flanders, Belgium** |
| **Investigator:** | **Christiana Nöstlinger**<br>**Department of Public Health** |
| | |
| Background and study objectives (rationale and relevance): | The study follows the previous research steps and is relevant. |
| Approach for the recruitment of the study subjects: | Sound |
| Study costs: | NA |
| Study procedures: | See additional comments |
| Risk evaluation: | There are no risks for GPs. The study uses routine HIV surveillance data, will be used for monitoring and evaluation. |
| Benefit evaluation: | Not relevant |
| Confidentiality and privacy: | Not relevant |
| Informed consent:<br>- language<br>- description of the content of the study<br>- foreseeable risks & benefits<br>- confidentiality<br>- privacy<br>- medical treatment<br>- contact information<br>- voluntary participation & refusal<br>- legal representative | Not relevant. |
| Final remarks: | There are no ethical issues with this study. The study does not need to be submitted to the EC of UZA. |
| **Conclusion** | **Favourable (= IRB approval)** |

**PART 2: Other comments for the investigators**
**(it is up to their discretion whether or not these are addressed)**

| | |
|---|---|
| Background and study objectives (rationale and relevance): | |
| Approach for the recruitment of the study subjects: | |
| Study procedures: | A structured self-administered questionnaire is a limited research method for a process evaluation including a feasibility and acceptability study. There is much more to do to yield interesting results. The IRB suggest the researchers to review their design regarding this. |
| Final remarks: | |
