# Supplementary material for: Development and Evaluation of an HIV-Testing Intervention for Primary Care: Protocol for a Mixed Methods Study
Source: JMIR Res Protoc. 2020 Aug 17;9(8):e16486. doi: 10.2196/16486 (PMC7459432; doi:10.2196/16486)
Supplement: Multimedia Appendix 2 [file resprot_v9i8e16486_app2.pdf]

Dr Virginie Supervie
INSERM U1136
Clinical epidemiology of HIV infection
56 Boulevard Vincent Auriol
75013 PARIS
FRANCE

Dear Dr Supervie,

Thank you again for submitting your proposal “HERMETIC” in response to the third transnational call of the ERA-Net HIVERA.

We are very pleased to inform you that your proposal has been recommended for funding. This recommendation is based on a scientific evaluation of the proposals by peer reviewers and a ranking list derived from these evaluations by the ERA-Net HIVERA Call Partnership Board. Please find enclosed a brief summary of the evaluation within the Scientific Evaluation Committee (SEC) and the Call Partnership Board.

It may be possible that subprojects of the consortium can only be funded with a reduced budget or not at all. To clarify this and discuss further procedures every subproject leader should contact his/her national funding organisation. Please inform your cooperation partners about the review results and encourage them to contact their national funding organisation.

The final funding decision will depend on national regulations and inspection of the formal proposals to be submitted to the national funding organisations. Each national funding agency will then take a formal decision on the projects to be supported. Please note that although there is a good chance that your proposal will be funded this letter does not guarantee funding. Furthermore, your project plan and budget still have to be approved by the national funding agencies. There will be no redress in law according to this message.

Yours sincerely,

HIVERA 2014 Joint Call Secretariat

On Behalf of the HIVERA Call Partnership Board

CC:
- Jean-François Delfraissy, ANRS France
- Liselotte De Vos, VLAGEW, Belgium
- UIdis Berkis, LAS, Latvia

Enclosure:

- Summary of the evaluation
